# Polyphenols and microglial dynamics in neurodegenerative diseases: mechanistic advances and therapeutic perspectives

**DOI:** 10.7717/peerj.21359

**Published:** 2026-07-27

**Authors:** Luping Liu, Huan Xiao, Jinhong Zhou, Muxi Zhang, Junguo Duan

**Affiliations:** 1 Chengdu University of Traditional Chinese Medicine, Chengdu, China; 2 Suining First People’s Hospital, Sui Ning, China; 3 Eye Health with Traditional Chinese Medicine Key Laboratory of Sichuan Province, Chengdu, China

**Keywords:** Polyphenols, Microglial dynamics, Microglia polarization states, Neuroinflammation, Neurodegenerative diseases

## Abstract

Neuroinflammation plays a central role in multiple neurological and neurodegenerative disorders, including ischemic brain injury, Alzheimer’s disease (AD), and Parkinson’s disease (PD). Microglia, the principal immune cells with in the central nervous system (CNS) are pivotal mediators of neuroinflammatory responses *via* their dynamic transition across a spectrum of polarization states, broadly delineated by pro-inflammatory M1-like and anti-inflammatory M2-like phenotypic profiles. A pathological skew towards pro-inflammatory microglial activation drives and exacerbates disease progression, thereby rendering the modulation of microglial polarization states a promising therapeutic target for neuroprotective intervention. Natural polyphenols have garnered increasing interest owing to their capacity to traverse the blood-brain barrier (BBB), confer neuroprotective effects, and mitigate neuroinflammation. Despite challenges in clinical translation stemming from poor bioavailability and rapid *in vivo* metabolism, innovative delivery systems are being developed to address these limitations. This review consolidates current evidence regarding the mechanisms by which polyphenols modulate microglial phenotypic balance and polarization states and examines advanced delivery strategies designed to enhance their therapeutic efficacy in neuroinflammatory disorders. By synthesizing these perspectives, we offer novel insights into the potential application of polyphenols in neuroprotective therapies targeting pathological neuroinflammation.

The target audience is researchers, clinicians, and students in the fields of neuroscience, pharmacology, and medicine, particularly those interested in neuroinflammation, microglial phenotypic balance and polarization states, and the neuroprotective effects of polyphenols.

## Survey methodology

Several pivotal steps were taken to guarantee the comprehensiveness and objectivity of this review. First, a systematic literature search on PubMed, Web of Science, and Scopus was performed for articles published from 2013 to 2025. The search terms used included “polyphenol”, “neuroinflammation”, “microglia”, “M1-like pro-inflammatory polarization state”, “M2-like anti-inflammatory/reparative polarization state” and their respective synonyms. “M1 polarization” and “M2 polarization” were also included to ensure comprehensive coverage of existing literature, as these oversimplified binary classifications remain prevalent in published studies. These terms were combined using Boolean operators (AND/OR) to capture all relevant studies.

Following database retrieval, we removed duplicate articles and implemented a two-stage screening protocol: initial triage on the basis of title/abstract relevance, followed by a full-text evaluation using predefined criteria: (1) inclusion of original research articles (*in vitro* or *in vivo*) and seminal reviews published in English; exclusion of conference abstracts, editorials, and non-peer-reviewed literature; (2) methodological rigor, e.g., appropriate controls and clear characterization of microglial phenotypic states; (3) prioritization of studies with direct relevance to the interplay between polyphenols and microglial phenotypic balance and polarization states. In addition, we manually searched the reference lists of key included articles to identify any additional pertinent publications, thereby maintaining the integrity of our review.

## Introduction

Neurological disorders have emerged as the foremost cause of disability and the second leading cause of mortality worldwide, presenting a substantial public health challenge that is further intensified by an aging population (Feigin et al., 2020). A unifying pathological feature across various conditions-such as cerebrovascular, degenerative, and psychiatric diseases-is chronic neuroinflammation. This sustained immune response within the central nervous system (CNS) plays a critical role in driving disease progression (DiSabato, Quan & Godbout, 2016; Brambilla, 2019).

Microglia (MG), the resident macrophages of the CNS, are pivotal in orchestrating this process. As the primary immune sentinels, their activation is characterized by considerable plasticity and is highly dependent on the context. Traditionally, this activation has been described as a dichotomy between a pro-inflammatory, neurotoxic M1 phenotype and an anti-inflammatory, reparative M2 phenotype (Wendimu & Hooks, 2022; Orihuela, McPherson & Harry, 2016). However, this binary perspective is now regarded as a conceptual oversimplification. It is well- established that microglia *in vivo* exhibit a continuous spectrum of functional polarization states, where M1-like and M2-like states represent merely two anchor points rather than discrete, mutually exclusive phenotypes. This dynamic spectrum reflects their remarkable adaptability to the CNS’s ever-changing microenvironmental cues (Guo, Wang & Yin, 2022; Weekman et al., 2014).

Beyond microglial phenotypic regulation, recent neuroinflammation research has uncovered critical new frontiers that expand our understanding of multi-component neuroinflammatory networks: ischemic preconditioning strategies can modulate endogenous neuroinflammatory responses to confer brain tissue protection; long-term sequelae of SARS-CoV-2 infection on olfactory neural networks have been linked to Alzheimer’s disease (AD) pathogenesis *via* dysregulated type I interferon signaling, which in turn disrupts microglial homeostatic polarization; astrocyte-specific mutations (*e.g*., LRRK2 G2019S) drive proinflammatory cytokine secretion and dopaminergic neurotoxicity in Parkinson’s disease (PD) by altering astrocyte-microglia crosstalk; and gut microbiota-targeted interventions can regulate microglial activation states and motor function in PD preclinical models (Di Santo et al., 2024; Vavougios et al., 2024; Ho et al., 2024; Panaitescu et al., 2024). These interconnected findings underscore the necessity of developing integrated therapeutic strategies that target multiple components of the neuroinflammatory regulatory network, rather than single cell populations or signaling pathways.

Current pharmacological approaches to managing neuroinflammation frequently demonstrate inadequate efficacy and are often limited by adverse side effects, highlighting the imperative for the development of safer and more efficacious therapeutic agents (Torquato et al., 2017). In this context, natural products, particularly polyphenols, emerge as a promising alternative. This extensive and structurally diverse class of compounds, prevalent in various foods and medicinal plants, is characterized by multi-target activity and favorable safety profiles (Nunes et al., 2020; Kiemlian Kwee, 2016). Notably, several key polyphenols, including quercetin, resveratrol, and baicalein, possess the ability to traverse the blood-brain barrier (BBB) (Kim et al., 2022). A substantial body of preclinical evidence corroborates their potent neuroprotective and anti-neuroinflammatory properties (Milke et al., 2018).

This review consolidates contemporary evidence regarding the capacity of polyphenolic compounds to modulate microglial phenotypic balance and polarization states as a core mechanism to mitigate pathological neuroinflammation. We delineate their overarching neuroprotective functions, elucidate the mechanisms of action of specific polyphenols, and explore innovative delivery strategies devised to address pharmacokinetic limitations. Additionally, we consider pertinent safety issues and translational challenges.

## The role of microglial polarization states in neuroinflammatory pathophysiology

In their quiescent state, MG act as immune sentinels, playing a crucial role in maintaining homeostasis within the CNS. Upon exposure to pathological stimuli such as ischemia, infection, or injury, MG undergo polarization into distinct phenotypes: the classically activated M1 state or the alternatively activated/acquired deactivated M2 state. Current understanding delineates that M2 polarization comprises several functional subtypes, namely M2a, M2b, and M2c. The M2a subtype, also known as “alternative activation,” is induced by interleukin-4 (IL-4) and IL-13 and is associated with tissue repair. The M2b subtype is involved in immunoregulation, whereas the M2c subtype, often referred to as “acquired deactivation,” is driven by IL-10 or transforming growth factor-beta (TGF-β) and is implicated in neuroprotection and tissue remodeling through mechanisms such as debris clearance and the resolution of inflammation (Walker & Lue, 2015; Yuan, Wu & Ling, 2019).

**Figure 1:**
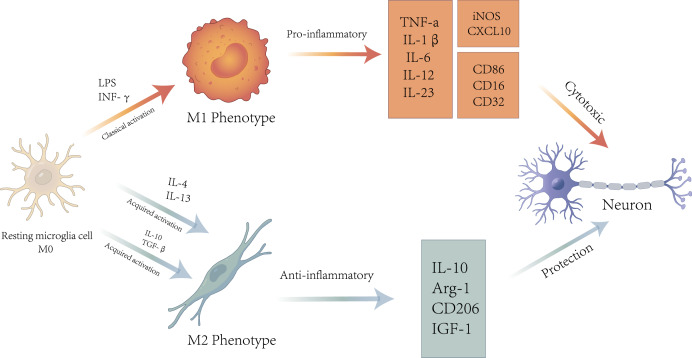
Two different polarization states of activated microglia. Schematic representation of M1 and M2 microglial phenotypes. Following activation, microglia undergo distinct morphological and functional polarization. M1-type microglia exhibit an amoeboid morphology and release pro-inflammatory mediators (*e.g*., IL-1β, TNF-α, iNOS), which exacerbate neuroinflammation and neuronal injury. In contrast, M2-type microglia display an elongated, ramified morphology and secrete anti-inflammatory cytokines (*e.g*., IL-10, TGF-β) and neurotrophic factors (*e.g*., IGF-1), promoting tissue repair and neuroprotection. Surface markers: M1 cells express CD16, CD32, and CD86; M2 cells express CD206 and Arg-1.

The M1 and M2 phenotypes are characterized by distinct differences in morphology, secretory profiles, and surface antigen expression (Tang & Le, 2016; Ronaldson & Davis, 2020). M1 microglia exhibit an amoeboid morphology and secrete pro-inflammatory mediators, including IL-1β, IL-6, TNF-α, inducible nitric oxide synthase (iNOS), and chemokine (C-X-C motif) ligand 10 (CXCL10), which contribute to the exacerbation of inflammation and neuronal injury. These cells also express surface markers such as CD16, CD32, and CD86. In contrast, M2 cells, when cultured *in vitro*, typically display an elongated and ramified morphology, release anti-inflammatory cytokines that promote repair and regeneration, and express markers such as CD206, arginase-1 (Arg-1), and neurotrophic factors, including insulin-like growth factor 1 (IGF-1) (Fig. 1).

### Mechanisms of microglial state transition in AD

In AD, the transition of microglial states represents a complex and multidimensional process that transcends the conventional M1 (pro-inflammatory) and M2 (anti-inflammatory) dichotomy. Advances in single-cell RNA sequencing have revealed a spectrum of disease-associated microglial states, such as disease-associated microglia (DAM) and microglia neurodegenerative phenotype (MGnD), underscoring their functional heterogeneity within AD pathology (Olah et al., 2020; Hashemiaghdam & Mroczek, 2020). This pathological state transition, often characterized by a progressive dysregulation of microglial phenotypic balance from a homeostatic reparative profile in early disease to a dominant pro-inflammatory profile in late-stage disease, is intricately regulated by cytokine signaling pathways. Key mediators, including IL-33, the NLRP3 inflammasome-IL-1β axis, IL-10, and the IL-12/IL-23 axis, play pivotal roles in modulating microglial function and influencing the progression of AD (Lau, Fu & Ip, 2021; Zhang et al., 2022). Notably, the absence of IL-10 promotes a pro-inflammatory M1 polarization (Laffer et al., 2019). In the early stages of AD, M2 microglia, activated by cytokines such as IL-4 and IL-10, perform neuroprotective functions, including the phagocytosis of amyloid-beta (Aβ). However, chronic exposure to Aβ eventually impairs these protective functions and fosters a deleterious M1 state, which exacerbates tau pathology, oxidative stress, and synaptic dysfunction (Cai et al., 2022; Balducci et al., 2017; Ayyubova, 2022; Rajendran & Paolicelli, 2018; Zhang et al., 2021a; Wang et al., 2023b).

### Mechanisms of microglial activation in PD

In PD, the fundamental pathological characteristics encompass the progressive degeneration of dopaminergic neurons within the substantia nigra pars compacta, alongside the formation of Lewy bodies, which are aggregates primarily composed of α-synuclein (α-syn) (Isik et al., 2023). Additionally, microglial activation and dysregulation of polarization states are initiated in the early stages of the disease and continue persistently as the disease advances (Lind-Holm Mogensen et al., 2025).

M1 Pro-inflammatory Polarization in PD: Misfolded α-syn fibrils function as damage-associated molecular patterns (DAMPs), effectively activating microglia through the TLR2 and NLRP3 inflammasome pathways (Harms, Ferreira & Romero-Ramos, 2021; Li et al., 2021). This activation initiates caspase-dependent apoptotic cascades, which directly contribute to the death of dopaminergic neurons. Furthermore, NLRP3 activation may enhance systemic immune priming (Jewell, Herath & Gordon, 2022). Pro-inflammatory stimuli further induce oxidative stress in microglia *via* ERK-dependent pathways, leading to the generation of reactive oxygen species (ROS) (Liu et al., 2020). Aberrant α-syn species exacerbate microglial activation while simultaneously impairing synaptic function and mitochondrial homeostasis (Wong & Krainc, 2017). Additionally, neuron-released aggregated α-syn disrupts microglial phagocytosis, resulting in defective clearance of protein aggregates and cellular debris, thereby accelerating neurodegeneration (Choi et al., 2015).

M2 Anti-inflammatory Polarization in PD: Anti-inflammatory/reparative microglial polarization states are predominantly elicited by anti-inflammatory cytokines such as IL-4, IL-10, IL-13, and TGF-β. Microglia exhibiting M2 polarization secrete factors such as Arg-1 and IGF-1, which facilitate tissue repair and matrix deposition. The pivotal regulator TREM2 serves a dual function in this process by promoting M2 macrophage polarization and inhibiting M1 macrophage activation through the suppression of PI3K/AKT, TLR, and MAPK signaling pathways. Concurrently, it facilitates phagocytic clearance and supports anti-inflammatory signaling (Yin et al., 2024). These mechanisms contribute to the attenuation of neuroinflammation and the protection of neuronal integrity. Preclinical investigations suggest that promoting microglial polarization towards the M2 phenotype, such as through IL-4 administration, mitigates motor deficits and confers neuroprotection in animal models of PD, highlighting the pathogenic role of deficient M2 polarization in the disease (Liu et al., 2024).

## Related signal pathways

**Figure 2:**
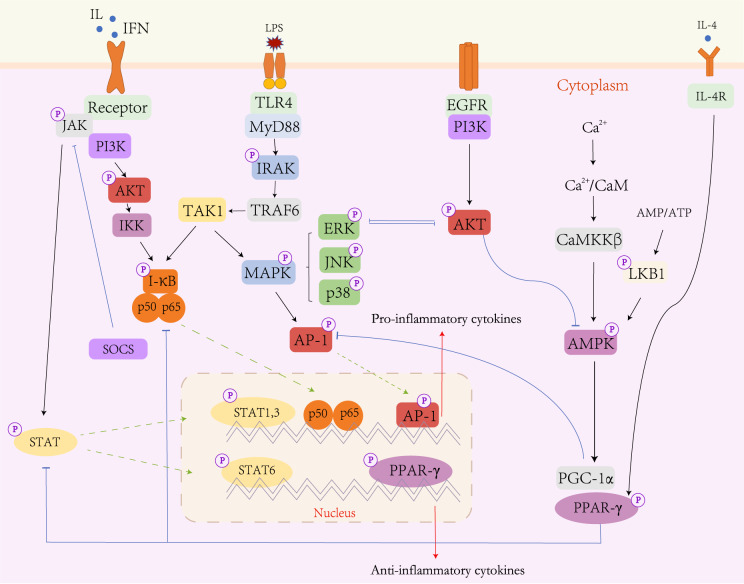
Key signaling pathways mediating microglia-induced neuroinflammation. Schematic illustration of major signaling cascades involved in microglial activation and neuroinflammation. Upon stimulation, multiple intracellular pathways are activated to regulate inflammatory responses. The MAPK cascade promotes the transcription of pro-inflammatory genes, while the JAK/STAT pathway mediates cytokine signal transduction. NF-κB serves as a central transcriptional regulator driving the expression of inflammatory mediators. Activation of TLR4 triggers the release of pro-inflammatory cytokines, exacerbating neuroinflammation and contributing to neuronal damage. In contrast, PPARγ activation inhibits NF-κB signaling, thereby suppressing pro-inflammatory mediator production and exerting neuroprotective effects.

Microglia phenotypic balance and polarization states are regulated by a network of evolutionarily conserved key signaling pathways that mediate neuroinflammatory responses in the CNS. The MAPK cascade is instrumental in promoting the transcription of inflammatory genes, whereas the JAK/STAT pathway is responsible for transducing cytokine signals. NF-κB functions as a central regulator of pro-inflammatory gene expression. Activation of TLR4 triggers the release of inflammatory cytokines, thereby exacerbating neuroinflammation and contributing to neurodegeneration. Conversely, activation of PPARγ has the potential to inhibit NF-κB, leading to a reduction in the production of pro-inflammatory mediators and conferring neuroprotective effects (Lim et al., 2018; Qu et al., 2019; Zhao et al., 2020; Li et al., 2020; Yang et al., 2020b; Zhou, Ji & Chen, 2020) (Fig. 2).

In recent years, significant progress has been made in understanding the mechanisms underlying microglial polarization. Beyond the well-characterized NF-κB and MAPK signaling pathways, emerging evidence has revealed novel mechanisms through which polyphenols modulate microglial activation.

(1) Immunometabolic reprogramming
  Metabolic reprogramming, notably the transition between glycolysis and oxidative phosphorylation, plays a pivotal role in regulating microglial polarization and the progression of disease. In the context of cerebral ischemia/reperfusion injury, the pathological microenvironment induces a shift in microglial metabolism towards glycolysis, thereby promoting a pro-inflammatory M1 phenotype that exacerbates neuroinflammation and tissue damage (Sun et al., 2023). This metabolic alteration is similarly observed in other neurological disorders; for instance, in Alzheimer’s disease, the mTOR-AKT-HIF-1α signaling pathway facilitates comparable metabolic reprogramming, which contributes to neuroinflammation and cognitive decline. Targeting these metabolic pathways, such as through the use of glutathione-functionalized gold nanocages, has shown promise in modulating microglial polarization and ameliorating disease symptoms (Yang et al., 2023).
(2) Epigenetic modification
  The regulation of microglial polarization through epigenetic mechanisms is a burgeoning field of research. For instance, the long noncoding RNA HOXA-AS2 has been shown to influence microglial polarization by recruiting the polycomb repressive complex 2 (PRC2) and modulating the histone methylation of the PGC-1α promoter. This interaction promotes M1 polarization and neuroinflammation in Parkinson’s disease, suggesting that targeting HOXA-AS2 could be a viable therapeutic strategy (Yang et al., 2021). In ischemic stroke, microglia and macrophages experience substantial epigenetic alterations that affect their polarization states. These modifications encompass histone methylation, DNA methylation, and non-coding RNA regulation, which collectively contribute to the pathophysiological processes underlying ischemic brain injury. Elucidating these mechanisms may facilitate the development of innovative diagnostic and therapeutic strategies for stroke (Qiu, Xu & Zhan, 2021).
(3) Inhibition of ferroptosis
  Ferroptosis has been implicated in various pathological conditions, including AD and cerebral ischemia-reperfusion injury, with its regulation intricately connected to microglial polarization. Notably, the knockout of estrogen-related receptor alpha (ERRα) promotes M2 microglial polarization and mitigates ferroptosis, highlighting its significance in modulating microglial function in sepsis-associated brain injury (Jin et al., 2025). Investigations into different agents further elucidate coordinated mechanisms: the traditional Chinese formula Naotaifang facilitates the transition of microglia from the M1 to M2 phenotype *via* the BMP6/SMADs signaling pathway, thereby reducing both inflammation and ferroptosis (Liao et al., 2023). Similarly, Schisandrin B inhibits neuronal ferroptosis and subsequent M1 polarization, leading to improvements in cognitive deficits associated with AD (Ding et al., 2025). Taken together, these findings suggest that targeting specific signaling pathways to concurrently regulate microglial polarization and ferroptosis may present novel therapeutic strategies for neurodegenerative disorders.
(4) Gut–brain axis
  The gut microbiota and its metabolites play a pivotal role in the regulation of microglial phenotypic balance and polarization states through the gut-brain axis. The composition of the intestinal flora is a key determinant of microglial activation, with specific genera, such as Ruminococcus, promoting an anti-inflammatory M2 phenotype (Ding et al., 2022). Microbial metabolites, particularly short-chain fatty acids like sodium butyrate, mitigate microglia-driven inflammation by modulating signaling pathways, including TLR4/MyD88/NF-κB, while also enhancing the integrity of the intestinal barrier (Sun et al., 2025). Additionally, natural compounds such as berberine and traditional formulations like Huanglian Wendan Decoction influence microglial polarization in a microbiota-dependent manner, often by stimulating the production of anti-inflammatory metabolites (Jalouli et al., 2025; Shi et al., 2025).

Recent mechanistic studies have further elaborated on these microbiota-mediated neuroprotective actions of natural products, identifying compound-specific modulatory effects on gut microbial communities: green tea polyphenols selectively enrich SCFA-producing bacterial taxa (*e.g*., Roseburia spp., *Faecalibacterium prausnitzii*) while reducing the abundance of pro-inflammatory pathogenic strains, thereby enhancing microbial-derived SCFA signaling to suppress microglial pro-inflammatory activation (Bárcena et al., 2019; Henning et al., 2018; Yuan et al., 2018). Curcumin remodels gut microbial ecosystems by shifting pro-inflammatory taxa toward beneficial commensal genera including Bifidobacterium and Lactobacillus, which in turn promote anti-inflammatory cytokine production to stabilize microglial phenotypic balance (Crovesy et al., 2017). Resveratrol modulates gut microbial metabolism to reduce trimethylamine N-oxide (TMAO) production—a pro-inflammatory metabolite linked to neurodegeneration—while improving systemic metabolic function, indirectly attenuating microglial neuroinflammatory signaling cascades (Rauf et al., 2018; Druart et al., 2014).

## Mechanisms underlying polyphenol-mediated modulation of neuroinflammation *via* regulation of microglial polarization states

Polyphenolic compounds, which are broadly categorized into flavonoids (such as flavonols and flavones) and non-flavonoids (such as phenolic acids and stilbenes), exhibit neuroprotective properties primarily by reducing neuroinflammation and neuronal apoptosis through the modulation of critical inflammatory signaling pathways. In addition to mitigating damage, these compounds actively enhance neuronal survival, synaptic plasticity, and cognitive function (Jalouli et al., 2025; Zhang et al., 2021b).

### Flavonoids

Flavonoids, also known as flavonoid compounds, typically refer to a series of compounds formed by the connection of two benzene rings (A ring and B ring) through a central three-carbon chain, that is, compounds with a C6-C3-C6 structure (Ku et al., 2020). Flavonoids are the most abundant phenolic compounds and are the active ingredients in most traditional Chinese medicinal materials, being more commonly found in plants of the Asteraceae, Fabaceae, and Apocynaceae families (Amiri et al., 2024). Most flavonoids exist in plants as glycosides combined with sugars, with only a few present in free form (Kotik, Kulik & Valentová, 2023). These compounds exhibit a variety of biological activities, mainly including anti-inflammatory, anti-tumor, antioxidant, and anti-mutagenic activities (Huang, Kim & Cho, 2023). Among them, galangin, quercetin, rutin, isovitexin, naringenin, baicalin, and anthocyanins have all been proven to have therapeutic effects against neuroinflammation.

#### Galangin

Galangin, a natural flavonol extracted from *Alpinia galanga*, exhibits promising therapeutic potential for addressing neuroinflammatory and neurodegenerative disorders, including stroke and cognitive impairment. This potential is primarily supported by preclinical evidence derived from cellular and rodent models (Yang et al., 2016; Wu et al., 2015; Kim et al., 2019) . The underlying mechanism involves the modulation of microglial phenotypic balance and polarization states through the activation of peroxisome proliferator-activated receptor γ(PPAR-γ), which is abundantly expressed in brain microglia and facilitates the promotion of an anti-inflammatory M2-like phenotype (Ding et al., 2020). Empirical studies indicate that galangin attenuates microglial activation and the expression of pro-inflammatory markers, while concurrently enhancing the expression of the anti-inflammatory cytokine IL-10, both *in vitro* and *in vivo* (Choi et al., 2017). Mechanistically, galangin activates PPAR-γ and Nrf2 signaling, thereby suppressing NF-κB-mediated pro-inflammatory gene expression. Importantly, the interaction between these pathways is highlighted by the negative regulation of Nrf2 by the NF-κB subunit p65, thus influencing neuroinflammatory and oxidative stress responses (Wardyn, Ponsford & Sanderson, 2015).

#### Quercetin

Quercetin, a major dietary flavonol present in vegetables, fruits, and various traditional Chinese medicinal herbs, is capable of traversing the BBB and demonstrates neuroprotective effects in the context of neurodegenerative diseases. These effects include the preservation of the blood-brain barrier, anti-inflammatory activities, and enhanced microcirculation (Alharbi et al., 2025; Li et al., 2023). Preclinical investigations have revealed its capacity to influence microglial polarization. *In vitro* studies indicate that quercetin reduces the expression of M1 markers and encourages an anti-inflammatory M2 phenotype through AMP-activated protein kinase (AMPK)/Akt signaling, thereby increasing IL-10 levels (Tsai et al., 2021). Importantly, quercetin mitigates the excessive phagocytic response of microglia to inflammatory stimuli, an effect interpreted as the restoration of homeostatic function rather than a broad immunosuppressive action. In a murine model of vascular dementia, induced by bilateral common carotid artery stenosis and chronic stress, quercetin administration ameliorates anxiety- and depression-like behaviors. This therapeutic effect is linked to increased secretion of anti-inflammatory cytokines, a shift towards M2 microglial polarization, reduced production of pro-inflammatory factors, and decreased hippocampal demyelination, synergistically leading to improved neuropsychiatric outcomes (Tan et al., 2022).

#### Rutin

Rutin, a flavonol glycoside abundant in various plants and traditional Chinese herbs, crosses the BBB and exhibits neuroprotective properties within the central nervous system. It shows potential in slowing neurodegenerative disease progression and improving cognitive function (Hosseinzadeh & Nassiri-Asl, 2014; Chunmei & Shuai, 2025). Within the CNS, Toll-like receptors (TLRs) are primarily expressed by MG. TLR-4 is a member of the TLR family. Overexpression of TLR4 often activates the TLR4 adaptor protein myeloid differentiation primary response gene 88 (MyD88), forming a MyD88-TLR4 complex that further activates NF-κB, thereby promoting the phenotypic transformation of MG (Yin et al., 2018). Lang, Li & Han (2021) pre-treated LPS-induced BV-2 cells with rutin and found that it may alleviate neuroinflammation by inhibiting the TLR4/NF-κB signaling pathway, thereby driving a shift in microglial phenotypic balance from pro-inflammatory to anti-inflammatory/reparative states. Further research is required to ascertain whether rutin’s influence on microglial polarization occurs through direct interaction with the TLR4/NF-κB signaling pathway or through the indirect modulation of the expression of its components.

#### Isovitexin

Isovitexin, a C-glycosyl flavone found in various plants, can cross the BBB and exhibits anti-inflammatory properties (He et al., 2016; Li et al., 2015). An increasing number of studies have indicated that AMPK plays a central role in neuroinflammation, with activated AMPK promoting M2 microglial polarization through various downstream pathways (Wang et al., 2018). Calcium/calmodulin-dependent protein kinase kinase-β (CaMKKβ) is an upstream activator of AMPK, and activation of the CaMKKβ/AMPK pathway can prevent microglia-mediated neuroinflammatory encephalopathy (Li et al., 2018). PGC-1α activation regulates the expression of genes involved in mitochondrial biogenesis and neuroinflammation. Research has demonstrated that isovitexin promotes M2 microglial polarization and exerts a potent inhibitory effect on LPS-induced neuroinflammation by activating the CaMKKβ/AMPK-PGC-1α signaling axis (Liu et al., 2020). These data suggest that isovitexin enhances M2 polarization *via* metabolic reprogramming of microglia.

#### Naringenin

Naringenin, a flavanone prevalent in citrus fruits, tomatoes, and various traditional Chinese medicinal herbs, has the capacity to traverse the BBB. Both dietary studies and preclinical research indicate that the consumption of naringenin may play a role in the prevention of chronic neurological disorders, such as depression and neurodegenerative diseases (Atoki et al., 2024; Lawal, Olotu & Soliman, 2018; Alam et al., 2014).

In AD, MG play a crucial role in the clearance of Aβ. The presence of Aβ induces the activation of M1-type microglia, which subsequently release pro-inflammatory cytokines, thereby exacerbating axonal degeneration and neuronal death. This process contributes to the disruption of neural networks, which is associated with memory impairment (Tang & Le, 2016). Evidence indicates that naringenin promotes the shift of MG to the M2 phenotype, thereby upregulating Aβ-degrading enzymes and inducing a reduction in Aβ plaques, improving cognitive function in AD mice (Yang, Kuboyama & Tohda, 2019). Additionally, it has been found that naringenin shifts microglial polarization towards the M2 state by inhibiting the activation of the MAPK signaling pathway, thereby alleviating neuroinflammatory responses (Zhang et al., 2019a).

#### Baicalin

Baicalin, a major flavone glycoside from Scutellaria baicalensis, exhibits potent neuroprotective activity (He et al., 2025). It efficiently traverses the blood-brain barrier, attaining significant concentrations in cerebrospinal fluid and exhibiting pronounced accumulation in critical brain regions, including the striatum, hypothalamus, and hippocampus (Ai et al., 2022; Wang et al., 2021).

The triggering receptor expressed on myeloid cells 2 (TREM2) is an innate immune receptor that is specifically localized on the membranes of MG and plays a critical role in modulating neuroinflammatory responses in the CNS (Wang et al., 2016). Studies have demonstrated that baicalin is capable of influencing the M1/M2 phenotypic balance, mitigating the imbalance between TREM2 and TLR4 in neuronal cells, and inhibiting the activation of downstream NF-κB signaling pathways to exert anti-neuroinflammatory effects (He et al., 2022).

#### Anthocyanins

Cyanidin-3-O-glucoside (C3G), the predominant anthocyanin present in foods such as black rice and blueberries, exerts neuroprotective effects in models of multiple neurodegenerative diseases (Ereminas et al., 2017; Ali et al., 2018; Skemiene et al., 2020; Fan et al., 2018). Upon absorption, anthocyanins are capable of traversing the BBB and accumulating in various brain regions, including the cortex, hippocampus, and striatum (Rashid et al., 2014). However, their clinical translation is constrained by challenges related to metabolic instability and rapid systemic clearance.

The progression of AD is associated with changes in TREM2 expression and weakened phagocytosis of Aβ by MG (Akhter et al., 2021; Wolfe et al., 2018). Studies by Sanjay et al. (2022) have shown that C3G regulates microglial phenotypic balance by activating PPARγ and enhancing the phagocytosis of Aβ to eliminate accumulated β-amyloid by upregulating TREM2.

### Nonflavonoids

Although resveratrol and curcumin, as prominent non-flavonoid polyphenols, possess distinct chemical structures and target different initial molecular pathways, they ultimately converge on two critical neuroprotective mechanisms. These mechanisms include the inhibition of the nuclear NF-κB-mediated inflammatory cascade and the enhancement of mitochondrial function, which collectively serve to mitigate oxidative stress and apoptosis.

#### Resveratrol

Resveratrol, a non-flavonoid polyphenolic compound within the stilbene class, is present in plants from the Polygonaceae, Fabaceae, Vitaceae, and Apiaceae families, including species such as *Polygonum cuspidatum*, *Cassia obtusifolia*, *Rheum palmatum*, as well as in grapes, blueberries, peanuts, raspberries, mulberries, and red wine. Notably, resveratrol is capable of crossing the BBB and exhibits potent neuroprotective effects (Andrade et al., 2018).

Empirical evidence suggests that PGC-1α plays a dual role by not only inhibiting LPS-induced M1 activation through the suppressing of NF-κB activity but also by facilitating the polarization of MG towards the M2 phenotype *via* activation of the STAT pathway (Kauppinen et al., 2013). Furthermore, resveratrol promotes M2 polarization of microglia by enhancing PGC-1α expression and suppressing NF-κB activity (Yang et al., 2017).

MicroRNAs (miRNAs), a category of endogenous non-coding RNAs, have been identified as critical therapeutic targets in CNS injuries, with miR-155 inhibition demonstrating beneficial effects in certain neuroimmune disorders (Butovsky et al., 2015). Studies reveal that resveratrol enhances the polarization of microglia to the M2 phenotype and attenuates neuroinflammation following cerebral ischemia by inhibiting miR-155 (Ma et al., 2020).

Silent information regulator 1 (SIRT1) a member of the sirtuin family, exhibits significant neuroprotective properties that are intricately linked to neuroplasticity (Jiao & Gong, 2020). Research by Tang et al. (2021) demonstrated that pre-treatment with resveratrol ameliorates cognitive impairment induced by sevoflurane in developing mice. This effect is potentially mediated through the modulation of the SIRT1/NF-κB axis in the hippocampus and the regulation of microglial polarization states, thereby mitigating neuroinflammation resulting from repeated sevoflurane exposure (Tang et al., 2021). Nonetheless, whether SIRT2 is involved in resveratrol-related neuroprotective effects remains to be clarified (Wu et al., 2020).

#### Curcumin

Curcumin, a polyphenol derived from Curcuma species, crosses the BBB and exerts neuroprotective effects in models of neurodegenerative and ischemic diseases (Zhu et al., 2014; Lo Cascio et al., 2021; Liu et al., 2017). However, its efficacy is affected by limited bioavailability and frequently relies on particular formulations.

AMPK activation is a major molecular switch that promotes M2 polarization of microglia. Research by Qiao et al. (2020) has confirmed that curcumin enhances M2 polarization of microglia through the CaMKKβ-dependent AMPK signaling pathway.

Prior research has demonstrated that TLR4 is primarily expressed in MG following brain injury, and the downregulation of TLR4 expression facilitates the transition from the M1 to the M2 phenotype, thereby ameliorating neuroinflammation post-traumatic brain injury (Rosciszewski et al., 2018; Yao et al., 2017). Consequently, the inhibition of TLR4 activation may represent a promising therapeutic target for mitigating neuroinflammation subsequent to subarachnoid hemorrhage (SAH). Furthermore, previous investigations have indicated that curcumin can markedly attenuate LPS-induced inflammation by modulating microglial M1/M2 polarization, reducing the imbalance between TREM2 and TLR4, and inhibiting the activation of downstream NF-κB (Zhang et al., 2019b). Additionally, research conducted by Gao et al. (2019) has found that curcumin alleviates neuroinflammation by inhibiting the TLR4/MyD88/NF-κB axis and promoting M2 polarization.

**Figure 3:**
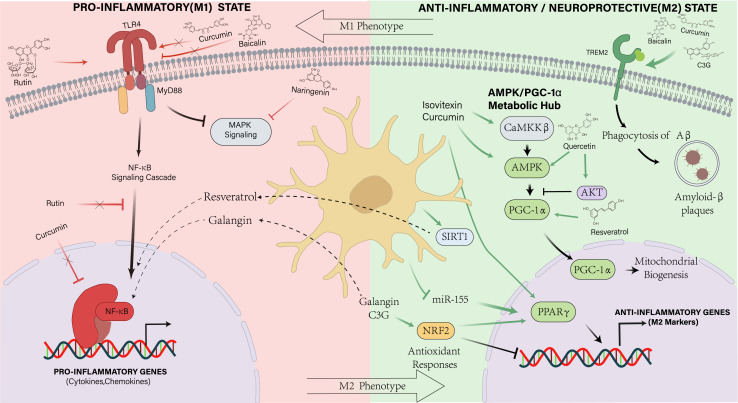
Polyphenols modulate the transition between pro-inflammatory M1 and anti-inflammatory/neuroprotective M2 phenotypes through multiple signaling pathways. Polyphenols regulate microglial polarization in neuroinflammation. (Left) In the pro-inflammatory M1 state, TLR4/MyD88-mediated MAPK and NF-κB signaling drives expression of pro-inflammatory genes. Polyphenols including rutin, curcumin, baicalin, and naringenin inhibit these pathways at multiple levels. (Right) In the antiinflammatory M2 state, polyphenols (isovitexin, curcumin, quercetin, resveratrol) activate the AMPK/PGC-1α metabolic hub to promote mitochondrial biogenesis. Additional mechanisms include TREM2 mediated Aβ phagocytosis (curcumin, baicalin, C3G), NRF2 antioxidant responses (galangin, C3G), and PPAR-γ-dependent anti-inflammatory gene expression, collectively driving microglial polarization toward the neuroprotective M2 phenotype.

To summarize, polyphenolic compounds alleviate neuroinflammation predominantly through the modulation of various critical signaling pathways, such as AMPK-PGC-1α activation, PPARγ induction, TLR4/NF-κB inhibition, and TREM2 signaling modulation. These coordinated signaling effects collectively act to restore homeostatic microglial phenotypic balance and regulate pathological polarization states. The detailed mechanisms of action for individual compounds are presented in Table 1 and Fig. 3.

**Table 1:** Polyphenol-mediated modulation of microglial phenotypic balance in neuroinflammation: mechanisms and molecular targets. Summary of polyphenolic compounds and their effects on microglial phenotypic balance and polarization states in various neuroinflammatory disease models. The table presents the active ingredients, disease models, experimental systems, and key molecular indicators of M1-like pro-inflammatory and M2-like anti-inflammatory/reparative polarization states. It should be noted that M1-like and M2-like states represent anchor points along a continuous spectrum of microglial functional polarization, rather than discrete, binary phenotypes. Upward arrows (↑) indicate upregulated markers associated with anti-inflammatory/reparative M2-like polarization state activation, including PPAR-γ, Nrf2, IL-10, CD206, Arg-1, YM1/2, TGF-β, and TREM2. Downward arrows (↓) indicate downregulated markers associated with pro-inflammatory M1-like polarization state suppression, including NF-κB, MAPK, JNK, PI3K/Akt, TLR4, NLRP3, iNOS, and pro-inflammatory cytokines (TNF-α, IL-1β, IL-6). LPS, lipopolysaccharide; IFN-γ, interferon-gamma; Aβ, amyloid-beta; BCAS, bilateral common carotid artery stenosis; CRS, chronic restraint stress.

Active ingredient	Disease model	Experimental model	Detection indicators
Galangin	Neuroinflammatory injury	Mice, BV2 cell line (LPS treatment)	PPAR-γ/Nrf2/CREB/IL-10 (↑), MAPK/JNK/PI3K/Akt/NF-κB/IL-6/TNF-α/iNOS (↓)
Quercetin	Neuroinflammatory injury	Adult mouse microglial cell line (LPS treatment)	IL-10/CD206/Akt/AMPK (↑), IL-1β/TNF-α/IL-6/CXCL10/iNOS (↓)
	Vascular dementia	BCAS/CRS mice, primary microglial cells	IL-10/IL-4/Arg-1/CD206 (↑), IL-1β/TNF-α/iNOS/CD46 (↓)
Rutin	Neuroinflammatory injury	BV2 cells (LPS treatment)	IL-10/CD206/Arg-1 (↑), TLR4/NF-κB/NLRP3/IL-6/TNF-α/IL-1β/CD86/iNOS (↓)
Isovitexin	Neuroinflammatory injury	Mouse primary cortical microglial cells, BV2 cells (LPS treatment)	PPARγ/PGC-1α/CaMKKβ/AMPK/Arg-1/CD206/YM1/2 (↑), TNF-α/IL-6/IL-1β/iNOS (↓)
Naringenin	Neuroinflammatory injury	BV2 cells (LPS treatment)	IL-4/IL-10/Arg-1 (↑), TNF-α/IL-1β/JNK/ERK1/2 (↓)
	Alzheimer’s disease	Primary cortical microglial cells (Aβ1–42 treatment)	CD206/Arg-1 (↑), M1/M2 ratio/CD16/CD32/iNOS (↓)
Baicalin	Neuroinflammatory injury	BV2 cells (LPS combined with IFN-γ treatment)	IL-4/IL-10/Arg-1/TREM2 (↑), IL-1β/IL-6/iNOS/TLR-4/NF-κB (↓)
Cyanidin-3-O-glucoside	Neuroinflammatory injury	APPswe/PS1ΔE9 mice, human microglial cell line (Aβ42 treatment)	CD206/CD163/Arg-1/PPARγ/TREM2 (↑), CD86/CD80/IL-1β/IL-6/TNF-α (↓)
Resveratrol	Neuroinflammatory injury	BV2 cells (LPS treatment)	PGC-1α/Arg-1/Ym1/TGFβ1/STAT3/STAT6 (↑), CD16/CD86 (↓)
	Neuroinflammatory injury	BV2 cells (LPS treatment)	IL-4/IL-10/CD206/Arg-1 (↑), miR-155/IL-6/IL-1/CD32/IL-1β (↓)
	Cognitive impairment	Mice (sevoflurane treatment)	CD206/SIRT1 (↑), CD86/SOCS3/NF-κB/IL-6/TNF-α (↓)
	Neuroinflammatory injury	BV2 cells (LPS treatment)	TREM2/Arg-1/IL-4/IL-10/CD206 (↑), TLR-4/iNOS/IL-1β/IL-6/CD16/32/NF-κB (↓)
Curcumin	Neuroinflammatory injury	BV2 cells, mouse primary cortical microglial cells (LPS treatment)	AMPK/CaMMβ/TNF-β/YM1/2/IL-10/Arg-1 (↑), TNF-α/IL-6/IL-1β/IL-2 (↓)
	Subarachnoid hemorrhage	tlr4–/– mice and wild type	CD206/IL-10/TGF-β (↑), CD86/IL-1β/IL-6/iNOS/TNF-α (↓)

## Drug delivery of polyphenolic compounds and associated challenges

### Limitations of polyphenol bioavailability

As previously mentioned, polyphenolic compounds have garnered widespread attention and application across numerous fields due to their diverse biological activities and ability to traverse the BBB. However, their clinical application remains constrained by several factors, including low solubility, inadequate permeability, instability, susceptibility to environmental influences, and limited bioavailability. To address these challenges, polyphenols are frequently encapsulated within various carriers, thereby improving biocompatibility, facilitating targeted release, and mitigating undesirable interactions *in vivo*. A range of delivery systems has been developed to date, encompassing lipid-based nanoparticles, protein-based nanoparticles, metal nanoparticles, nanoemulsions, nanofibers, liposomes, phospholipid complexes, polymers, micelles, and emulsions (Yang et al., 2020a; Andrade et al., 2018; Xu et al., 2023; Cui, Lin & Zhang, 2025).

### Advances in nanocarrier delivery

The BBB poses a significant challenge to the clinical delivery of therapeutic agents for central nervous system disorders, often necessitating invasive methods such as intracerebral implantation or intraventricular injection, both of which entail considerable risks, including infection (Chiang et al., 2025).

Nose-to-brain drug delivery (NBDD) has been identified as a promising non-invasive alternative. This approach primarily exploits the olfactory and trigeminal pathways to circumvent the BBB, facilitating rapid drug transport to the cerebrospinal fluid through intranasal administration and thereby markedly enhancing brain bioavailability (Xu et al., 2025). The effectiveness of this approach has been substantiated through preclinical studies. For example, one study developed an inhaled nanocatalytic therapy, demonstrating that intranasally administered Mn/Co₃O₄ nanoparticles can traverse the brain *via* similar pathways, target inflammatory regions in ischemic tissue, scavenge reactive oxygen species, and restore mitochondrial function, thereby alleviating cerebral ischemia (Wang et al., 2023a). Additionally, the encapsulation of baicalin into polyethylene glycol-polylactic acid-co-glycolic acid nanoparticles (PEG-PLGA NPs) and their administration *via* intranasal inhalation, resulted in significant alleviation of neurological deficits, reduction in cerebral infarction area, and mitigation of neural trauma and swelling in rats with ischemic brain injury (Pimentel-Moral et al., 2018).

In recent years, significant advancements have been made in the development of polyphenol delivery systems, as evidenced by the emergence of targeted nanoplatforms such as metal-phenolic networks, biomimetic carriers, engineered exosomes, and hydrogels. These innovations aim to enhance delivery efficiency, bioavailability, and lesion-specific accumulation of polyphenols, thereby improving their ability to modulate neuroinflammatory processes (Ozkan et al., 2020; Wu et al., 2019; Wang et al., 2019; Li et al., 2022). However, despite these advancements, the application of nanocarriers presents significant safety concerns.

### Toxicological concerns and model limitations

While nanocarriers have demonstrated potential in addressing neuroinflammation, their clinical application is hindered by significant challenges, notably nanotoxicity and the lack of standardized models. In contrast to organic nanomaterials, inorganic nanomaterials, such as gold nanoparticles and quantum dots, exhibit considerable resistance to degradation, potentially resulting in their prolonged accumulation within the brain. Recent investigations have indicated that these materials can disrupt the biosynthesis of microglial exosomes by inhibiting the ERK1/2 pathway, subsequently impeding their clearance *via* the perivascular glymphatic pathway and extending their cerebral retention (Gao et al., 2024). The interaction between nanocarriers and the nervous system can elicit toxicity through various mechanisms, including direct cellular damage and organelle interactions (Abbasi et al., 2023; Ramos et al., 2022). Oxidative stress emerges as a central mechanism, capable of inducing apoptosis, inflammatory cascades (Teixeira et al., 2024; Gandhi et al., 2024; Hsiao et al., 2016), endoplasmic reticulum stress (Dąbrowska-Bouta et al., 2022; Chojnacka-Puchta et al., 2025), and mitochondrial dysfunction (Yao, Zhang & Tang, 2024). For example, titanium dioxide nanoparticles have been shown to provoke oxidative stress and apoptosis in the hippocampus, leading to deficits in motor and spatial memory in rodent models (Czajka et al., 2015; He et al., 2018).

Insufficient standardization of disease models severely compromises the reliability and reproducibility of studies investigating polyphenol-based nanoformulations for microglial-polarization-directed therapy of neuroinflammation. The principal deficiencies are: (1) Species disparity. Critical differences in cellular, genetic, immunological and molecular features between humans and rodents limit the capacity of animal models to screen therapeutics that effectively target the human brain (Aday et al., 2016; Hajal et al., 2018). At present, there exists a notable deficiency of microglial cell models derived from human-induced pluripotent stem cells (iPSC), which constitutes a considerable technical constraint in contemporary research and simultaneously indicates a pathway for the development of more human-relevant research systems in the future. (2) Heterogeneity of neuroinflammation-induction protocols. Commonly used triggers-bacterial LPS, recombinant cytokines (*e.g*., TNF-α, IL-1β), or disease-specific stimuli such as Aβ in Alzheimer’s models-activate distinct microglial phenotypic profiles and inflammatory signaling pathways (Skrzypczak-Wiercioch & Sałat, 2022; Castellanos et al., 2024; Geloso, Zupo & Corvino, 2024). (3) Over-simplified BBB models. Most *in vitro* BBB platforms fail to recapitulate the intricate neurovascular unit, lacking the multicellular interplay among pericytes, astrocytes and microglia (Yin & Wang, 2024). A recent study revealed that brain endothelial-derived CCL17 engages CCR4 on perivascular astrocytes, promoting astrocytic C3 production that confers protection against nanoparticle-induced neurotoxicity (Ogaki et al., 2025). Recent advancements in organ-on-chip and 3D co-culture technologies have emerged as promising alternatives to overcome these limitations (Brown et al., 2015; O’Laughlin et al., 2025).

In conclusion, nanotechnology-based systems greatly enhance polyphenol delivery and therapeutic efficacy but raise new challenges related to safety, biodistribution, and model relevance. Integrating advanced *in vitro* systems and standardized toxicity protocols is critical for future clinical translation.

## Conclusion and future perspectives

Critical limitations in the preclinical evidence synthesized here must be acknowledged to contextualize polyphenol-based neuroprotective strategies. Virtually all mechanistic insights into polyphenol-mediated microglial phenotypic balance regulation stem from reductionist *in vitro* models (LPS-stimulated BV2 cells or rodent primary microglia), which fail to recapitulate *in vivo* microglial spatiotemporal heterogeneity shaped by brain niche cues and disease progression. Emerging single-cell RNA sequencing and spatial profiling studies have uncovered complex microglial phenotypic states (including DAM and stage-specific transitional populations) beyond the oversimplified M1/M2 framework, underscoring the need for physiologically relevant preclinical models (hiPSC-derived microglia, CNS organoids) to bridge the translational gap.

Although significant progress has been made, several critical challenges necessitate resolution through interdisciplinary collaboration:

(1) Mechanistic Complexity and Research Needs
  The mechanistic complexity and research demands associated with polyphenolic compounds are significant, as these compounds frequently display multi-component and multi-target properties, interacting with complex signaling networks that obscure the delineation of specific pathways. Future research should integrate the holistic framework of traditional Chinese medicine with modern molecular and pharmacological approaches. Additionally, the regulatory mechanisms governing microglial phenotypic balance and polarization states are intrinsically complex. For multifaceted pathologies, such as neurodegenerative diseases, employing a combination of regulatory approaches may result in more effective therapeutic strategies.
(2) Integration of Nanotechnology with Safety Considerations
  Nanotechnology provides an ideal carrier system for improving the pharmacokinetics and bioavailability of polyphenols. However, before clinical application, it is still necessary to consider minimizing their toxicity and side effects. Standardized *in vitro* and *in vivo* models, along with robust safety testing, are essential to advance nanoparticle development. Regulatory frameworks for nanoparticle-based therapeutics must also be developed to ensure safety and reproducibility.
(3) Translation to Clinical Research
  Most research on signaling pathways governing polyphenol-mediated regulation of microglial polarization states remains confined to preclinical studies, underscoring the need for translation into clinical settings. Well-designed clinical trials are required to validate preclinical findings.

In conclusion, polyphenols represent a promising class of multitarget neuroprotective agents, but their translation to the clinic requires integrated efforts in pharmacology, nanotechnology, regulatory science, and clinical neuroscience. Future research should also leverage emerging insights into neuroinflammation frontiers, such as preconditioning strategies, astrocyte-mediated neuroinflammation, and gut-brain axis interactions, to develop more targeted and effective polyphenol-based therapeutic approaches for neurological disorders.

## Additional Information and Declarations

### Data Availability

The following information was supplied regarding data availability:

This is a literature review.
